# Public health round-up

**DOI:** 10.2471/BLT.21.011021

**Published:** 2021-10-01

**Hypertension burden shifts**

A health volunteer measures the blood pressure of a patient during a home visit on 23 September 2020, in Bang Phut, Thailand. According to the first comprehensive global analysis of trends in hypertension prevalence, over the past 30 years the burden of this disease has shifted from high-income to low- and middle-income countries. The study also indicates that some 720 million people with hypertension do not receive the treatment they need.

**Figure:**
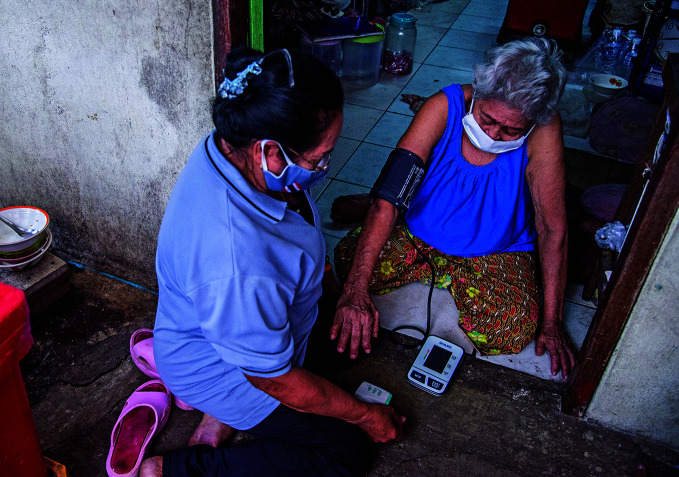
WHO/Ploy Phutpheng

## Afghan health services cut

A pause in funding for the Sehatmandi Project – the main source of health service delivery in Afghanistan – risked depriving millions of Afghans of access to essential health care.

According to a 6 September report by the World Health Organization Regional Office for the Eastern Mediterranean (EMRO), the funding pause went into effect in late August 2021, threatening to shut down 90% of the 2309 health facilities run by the project. Essential health care activities were already being interrupted by conflict and insecurity prior to the pause.

Dr Ahmed Al-Mandhari, EMRO Regional Director, acknowledged the constraints faced by donors but stressed the urgent need to identify a flexible funding mechanism for the continuation of the Sehatmandi Project which in 2020 delivered care to an estimated 30 million people and vaccinated 1.5 million children.

As part of a United Nations (UN) appeal for Afghanistan launched on 5 September, WHO and health sector partners are requesting US$ 66 million to deliver essential and life-sustaining health care services for 3.4 million people until the end of the year. As of 14 September, donors had pledged more than US$ 1.1 billion in response to the UN appeal, well in excess of the US$ 600 million originally targeted.

https://bit.ly/3lcpZ6Q

https://reut.rs/393VL0j

## WHO medical supplies arrive in Afghanistan

A shipment of medical supplies sent by the World Health Organization (WHO) to Afghanistan arrived at Kabul airport on 13 September. The shipment was among the first humanitarian aid to arrive at Kabul airport since operations were disrupted on 15 August and was flown by a Qatar Airways flight donated by the Government of Qatar. A second flight donated by Qatar was expected to arrive later in the week, carrying more WHO medical supplies.

The two shipments, which contain essential medicines such as insulin, medical consumables, trauma and surgery kits, and coronavirus disease 2019 (COVID-19) testing kits, will address the urgent health needs of 1.45 million people and provide for 5400 major and minor surgeries. They will be distributed to 280 health facilities and 31 public COVID-19 laboratories across Afghanistan.

https://bit.ly/3llIKoB

## Meningitis outbreak in the Democratic Republic of the Congo

The Democratic Republic of the Congo declared an outbreak of meningitis in the north-eastern Tshopo Province. Confirmatory tests carried out by the Institut Pasteur in Paris detected *Neisseria meningitidis* – one of the most frequent types of bacterial meningitis with the potential to cause large epidemics. As of 8 September, 261 people had been reported as infected, 129 of whom had died, representing a case fatality ratio of just under 50%. As of the same date, the health authorities had deployed an emergency team and, with WHO support, were quickly ramping up the response.

https://bit.ly/3k53ZLS

## New SARS-CoV-2 variant of interest

WHO classified the B.1.621 strain of severe acute respiratory syndrome coronavirus 2 (SARS-CoV-2) to be a variant of interest (VOI) on 30 August. Named Mu, the variant contains multiple mutations that indicate a potential for immune escape (the capacity to evade immune system response).

A VOI classification indicates a SARS-CoV-2 variant with genetic changes that are predicted or known to affect virus characteristics such as transmissibility, disease severity, and immune escape, and that have been identified as causing significant community transmission with increasing prevalence over time.

https://bit.ly/3A2x68h

## COVAX delivery challenge

COVAX forecast a likely shortfall in projected vaccine delivery for 2021. In a joint statement issued on 8 September, COVAX partners reported that, in the absence of urgent action by producers and high-vaccine-coverage countries, only 1.425 billion doses of vaccine will be made available in 2021, 575 million doses short of the two billion target set for this year. The two billion dose target is now expected to be reached in the first quarter of 2022.

Of the 1.425 billion doses, approximately 1.2 billion will be available for the lower-income economies participating in the COVAX Advance Market Commitment (AMC), enough to protect only 20% of the population in all 92 AMC-eligible economies, with the exception of India.

The vaccines pillar of the Access to COVID-19 Tools (ACT) Accelerator, COVAX is the only global initiative working with governments and manufacturers to ensure COVID-19 vaccines are available worldwide.

https://bit.ly/3Ecgxcx

## Outbreak surveillance hub opens

A new outbreak surveillance hub was opened in Berlin, Germany on 1 September. Part of WHO’s Health Emergencies Programme, the hub will tap the expertise of partners from a range of disciplines, supported by the latest technologies, to increase the availability of key data, develop analytic tools and predictive models for risk analysis, and link communities of practice around the world.

Critically, the hub will support the work of public health experts and policy-makers in all countries with the tools needed to forecast, detect and assess epidemic and pandemic risks so they can take rapid decisions to prevent and respond to future public health emergencies.

The hub is receiving an initial investment of US$ 100 million from the Federal Republic of Germany.

https://bit.ly/3zbtVK1

## WHO logistics delivers

The WHO Logistics Hub in Dubai, United Arab Emirates (UAE) delivered 85 metric tonnes of medical supplies to Ethiopia on 10 September, the largest single shipment of humanitarian cargo to date airlifted by the hub. The supplies, including essential medicines, trauma and emergency surgery kits, infusions, consumables, equipment, and cholera kits, were flown by a charter flight donated by the UAE.

The shipment concluded a historic week for the hub which shipped over 450 metric tonnes of medical supplies for the cholera outbreak response in Nigeria, medicines to Afghanistan, and trauma and surgical supplies to Syria and Yemen.

https://bit.ly/392ggKL

## Hypertension shift

The number of adults aged 30–79 years with hypertension increased from 650 million to 1.28 billion in the past 30 years. This is according to the first comprehensive global analysis of trends in hypertension prevalence, detection, treatment and control, led by Imperial College London and WHO, which was published on 25 August in The Lancet.

A key finding of the study was that, while there has been little change in the overall rate of hypertension in the world from 1990 to 2019, the burden has shifted from high-income to low- and middle-income countries. The rate of hypertension has in fact decreased in high-income countries – which now typically have some of the lowest rates.

Hypertension significantly increases the risk of heart, brain and kidney diseases. Around half of people living with hypertension are unaware of their condition, despite its being easily detected at home or in a health centre.

https://bit.ly/2Y6z6hz

## New technologies for priority diseases

WHO released a new compendium of 24 innovative health technologies for COVID-19 and other priority diseases that can be used in low-resource settings. Launched on 31 August, the compendium includes full assessments of the technologies by international experts working with WHO technical teams. The technologies are assigned a traffic-light score, indicating suitability or unsuitability for use, providing vital information to help governments, nongovernmental organizations and funders decide which products to procure.

https://bit.ly/2Xiu2qt

**Cover photo**

A young girl at the Shahrak-Sabz settlement for internally displaced people, Afghanistan. Since the start of the year more than 550 000 people, half of them children, have been internally displaced, fleeing drought and conflict in the country.

**Figure:**
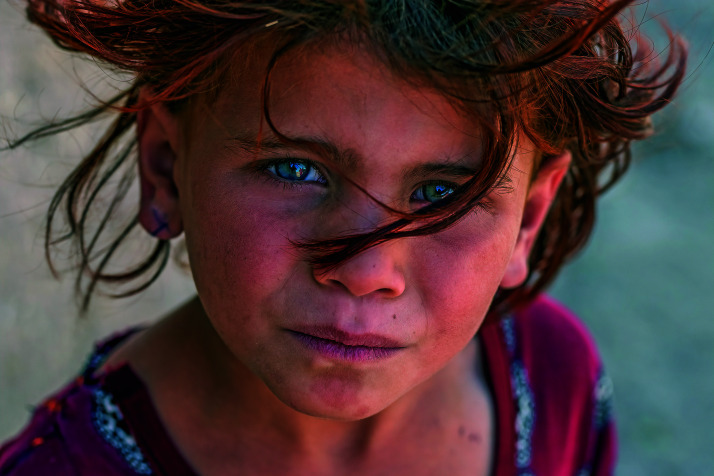
UNICEF//UN0509166/Bidel

## Dementia commitment challenge

Only a quarter of countries worldwide have a national policy, strategy or plan for supporting people with dementia and their families. This is according to WHO’s *Global status report on the public health response to dementia*, released on 2 September. Half of the countries are in WHO’s European Region, Even in Europe, many dementia plans are expiring or have already expired, indicating a need for renewed commitment from governments.

According to the report, more than 55 million people (8.1% of women and 5.4% of men over 65 years) are living with dementia, a number expected to rise to 78 million by 2030 and to 139 million by 2050.

https://bit.ly/2XnW6Zr

**Looking ahead**

21–28 October. FIGO World Congress. https://bit.ly/2YRr3FX

24–26 October. World Health Summit. https://bit.ly/33Np5W3

31 October–12 November. United Nations Climate Change Conference COP26. https://bit.ly/39hy1Gl

